# MicroRNAs in Alzheimer’s Disease: Function and Potential Applications as Diagnostic Biomarkers

**DOI:** 10.3389/fnmol.2020.00160

**Published:** 2020-08-21

**Authors:** Wei Wei, Zhi-Yong Wang, Li-Na Ma, Ting-Ting Zhang, Yu Cao, Hao Li

**Affiliations:** ^1^First Clinical College, Shandong University of Traditional Chinese Medicine, Jinan, China; ^2^Department of Geriatrics, Xiyuan Hospital, China Academy of Chinese Medical Sciences, Beijing, China

**Keywords:** microRNAs, Alzheimer’s disease, pathogenesis, diagnosis, biomarker

## Abstract

Alzheimer’s disease (AD) is the most common form of dementia. Although the incidence of AD is high, the rates of diagnosis and treatment are relatively low. Moreover, effective means for the diagnosis and treatment of AD are still lacking. MicroRNAs (miRNAs, miRs) are non-coding RNAs that play regulatory roles by targeting mRNAs. The expression of miRNAs is conserved, temporal, and tissue-specific. Impairment of microRNA function is closely related to AD pathogenesis, including the beta-amyloid and tau hallmarks of AD, and there is evidence that the expression of some microRNAs differs significantly between healthy people and AD patients. These properties of miRNAs endow them with potential diagnostic and therapeutic value in the treatment of this debilitating disease. This review provides comprehensive information about the regulatory function of miRNAs in AD, as well as potential applications as diagnostic biomarkers.

## Introduction

Alzheimer’s disease (AD) is the most frequently occurring dementia in the elderly. It is a multifactorial and heterogeneous neurodegenerative disease, clinically manifested as progressive cognitive dysfunction and behavioral impairment (Lane et al., 2018). The typical pathological features are essentially present with amyloid plaques and neurofibrillary tangles that are associated with beta-amyloid (Aβ) metabolism and the hyperphosphorylation of tau protein, respectively, as the core pathological mechanisms. Moreover, AD pathogenesis is closely related to impaired synaptic plasticity, immune-inflammatory responses, and numerous other processes associated with the central nervous system (CNS). MicroRNAs (miRNAs, miRs) are abundantly present in the CNS, and involve in the complicated pathogenesis of AD through a variety of mechanisms. The diagnosis of AD has great limitations currently. There is an urgent need for reliable biomarkers, especially in the early stages of the disease so that interventions can be promptly instituted to improve clinical outcome.

**FIGURE 1:**
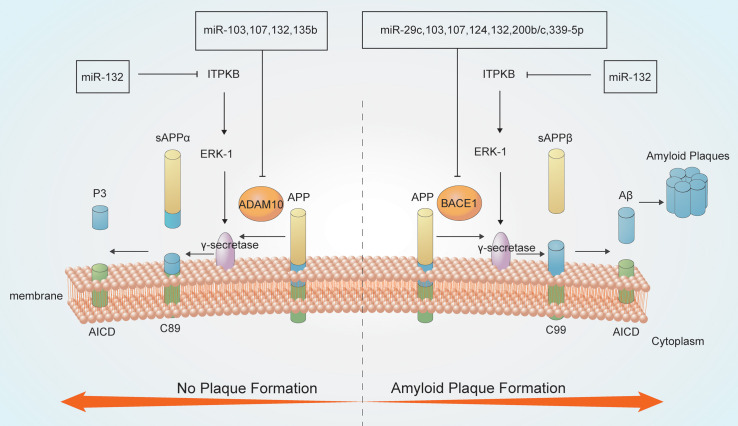
MicroRNAs (miRNAs) are involved in Aβ metabolism. Amyloid precursor protein (APP) is a type I integral inner membrane-localized protein. Under normal conditions, APP is hydrolyzed by α-secretase to produce the neuroprotective soluble external functional fragments (sAPP), P3 and the APP intracellular domain (AICD) (no plaque formation); in contrast, β-secretase-mediated APP hydrolysis generates plaque-forming Aβ, which is neurotoxic. The γ-secretase enzyme is crucial for both secretase pathways.

**FIGURE 2:**
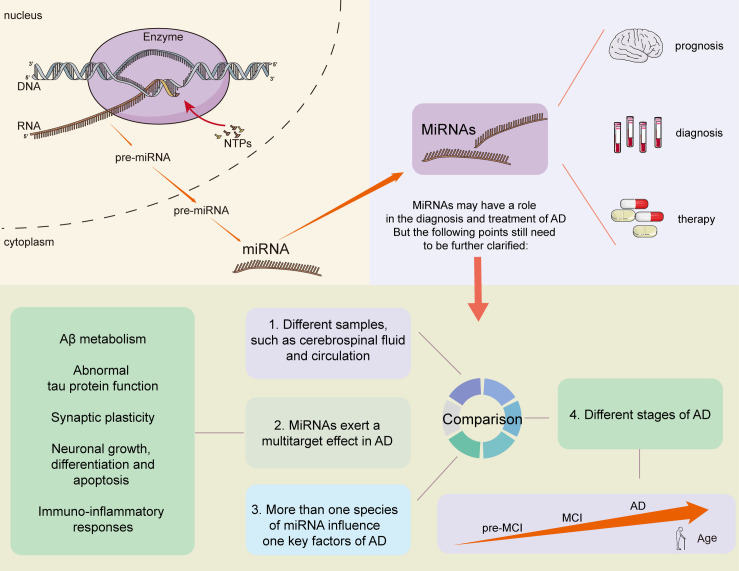
MiRNAs play a role to diagnose AD.

In this review, we summarize the evidence relating to how miRNAs modulate the onset and pathological progression of AD. Part of the summary is shown in Figure 1 and Table 1. We also review the potential of using miRNAs as diagnostic biomarkers for AD (Table 2), thereby providing a perspective of the clinical applications of miRNAs for AD management (Figure 2).

**TABLE 1:** Mysregulation of miRNAs in AD.

Species of miRNA	Changes	Materials	Targets and responses	References
miR-101	↓	HeLa cells	Regulates APP expression specifically via site 1 in 3′-UTR	Long and Lahiri, 2011
miR-106b	↓	SH-SY5Y cells	Fyn; tau hyperphosphorylation ↓	Liu et al., 2016
miR-124-3p	↓	HCN-2 cells and APP/PS1 mice; N2a/APP695swe cells	CAPN1; Caveolin-1-PI3K/Akt/GSK3β pathway tau hyperphosphorylation ↓	Kang et al., 2017; Zhou et al., 2019
miR-125b	↑	Primary hippocampal neurons; Neuro2a APPSwe/Δ9 cells; Human fatal cortical tissues;	Bcl-W, DUSP6, PPP1CA,FOXQ1; CDK5/p35/25; tau hyperphosphorylation ↑	Banzhaf-Strathmann et al., 2014; Ma et al., 2017; Jin et al., 2018
miR-128	↑	3×Tg-AD mice Neuro 2a (N2a) cells	PPARγ↓	Geng et al., 2018
miR-132	↓	the hippocampus of APP/PS1 mice	ITPKB; ERK1/2; BACE1; Aβ↑ tau hyperphosphorylation ↑	Salta et al., 2016
miR-132/miR-212	↓	APP/PS1 mice and HEK 293 cells; miR-132/212 knockout (KO) mice;	tau mRNA NOS1; tau hyperphosphorylation ↑	Smith et al., 2015; Salta and De Strooper, 2017; Wang et al., 2017
miR-135a	↑	AppTg (APPswe/PS1/E9 bigenic) mice U373MG cells	CCAA T/enhancer binding protein delta (CEBPD) thrombospondin 1 (THBS1) CEBPD/miR135a/THBS1 axis	Ko et al., 2015
miR-135b	↓	Primary hippocampal cells of senescence-accelerated mouse resistant 1 (SAMR1) mice	BACE1 ↓	Zhang B. et al., 2016
miR-137	↓	APP/PS1 mice and SH-SY5Y cells	CACNA1C; tau hyperphosphorylation ↓	Jiang et al., 2018
miR-138	↑	N2a/APP and HEK293/tau cells	RARA/GSK-3βsignal pathway; tau hyperphosphorylation ↑	Wang X. et al., 2015
miR-139	↑	The hippocampus of SAMP8 mice and primary hippocampal cells	Modulate CB2-meditated neuroinflammatory processes.	Tang et al., 2017
miR-140-5p	↑	Two regions of post-mortem brain (cerebellum and hippocampus)	ADAM10; SOX2; Aβ↑	Akhter et al., 2018
miR-146a	↑	SH-SY5Y cells and 5×FAD mice	ROCK1/PTEN signal pathway; tau hyperphosphorylation ↑ low-density lipoprotein receptor-related protein-2 (Lrp2) ↓ Lrp2/Akt pathway cell apoptosis	Wang et al., 2016; Zhang B. et al., 2016
miR-15/107 family (miR-103 and miR-107)	↓	SK-N-BE cells and HEK-293 cells post-mortem frozen brain tissue samples of AD patients	CDK5R1/p35; Aβ↑	Moncini et al., 2017
miR-107	↓	Cerebral cortex of AD patients; SH-SY5Y cells;	BACE 1 mRNA; ADAM 10; APP metabolism	Nelson and Wang, 2010; Augustin et al., 2012; Goodall et al., 2013
miR-153	↓	HeLa cells	APP, APLP2;	Long et al., 2012
miR−186	↓	AD rat models; hippocampal neuronal cells	Interleukin−2 (IL2) ↓; suppress the JAK–STAT signaling pathway	Wu et al., 2018
miR-188-5p	↓	5×FAD mice and primary hippocampal neuron	Synaptic dysfunction	Lee et al., 2016
miR-19	↓	SH-SY5Y cells	PTEN ↑ phosphorylated AKT ↓ p53 and Bax ↑ Bcl-2 ↓ neural cell apoptosis	Zhu M. et al., 2016
miR-200b/c	↑	APP/PS1 mice and PC12 cells; murine primary neurons SH-SY5Y cells	Ribosomal protein S6 kinase B1 (S6K1), PTEN; PI3K/mTOR signal pathway; Aβ↓	Wu et al., 2016; Higaki et al., 2018
miR-219	↓	Drosophila model that produces human tau SH-SY5Y cells and PC12 cells	MAPT; tau protein production ↓	Santa-Maria et al., 2015
miR-219-5p	↓	APP/PS1 mice and SH-SY5Y cells	TTBK1; Gsk-3β; tau hyperphosphorylation ↑	Li et al., 2019
miR-221	↓	SH-SY5Y cells	ADAM10 ↑	Manzine et al., 2018
miR-29c	↓	Hippocampus and the frontal cortex of the APPse/PSΔE9 mouse brain	BACE1; PKA/CRE signal pathway; Aβ↑	Zong et al., 2015
miR-302	/	SK-N-MC cells	*PTEN* Akt signaling Aβ-induced apoptosis	Li et al., 2016
miR-322	↑	The hippocampus of Tg2576 AD transgenic mouse	BDNF-TrkB; tau hyperphosphorylation ↑	Zhang et al., 2018
miR-33	↑	Brain of miR-33 (−/−) mice and primary neural cells	ABCA1; Metabolism of ApoE and Aβ; Aβ↑	Kim et al., 2015
miR-330	↓	AD mouse and primary neuron cells	VAV1; MAPK signal pathway; Aβ↑	Zhou et al., 2018
miR-339-5p	↓	heLa cells and U373 MG cells	BACE1; Aβ↑	Long et al., 2014
miR-34a	↓	APP/PS1 Tg mice and primary neural cells	Cyclin D1 ↑ regulate cell cycle apoptosis	Modi et al., 2016
miR-34c	↑	C57BL/6JNarl (B6) mice hippocampal primary cells	Influences dendritic spine density and synaptic plasticity	
miR-431	/	Cortico-hippocampal cells isolated from 3×Tg-AD mice	Kremen1 (Krm1); Wnt/β-cateninsignal pathway; regulate neurite outgrowth and synapse formation;	Ross et al., 2018
miR-4487	↓	SH-SY5Y cells	Cell apoptosis	Hu et al., 2018
miR-511	↓	HEK293T IMR-32 cells M17 cells Neuro-2a cells SH-SY5Y cells and HeLa cells	3′UTR of *FKBP5* neuronal differentiation	Zheng et al., 2016
miR-603	↑	HEK293 cells and HeLa cells	*LRPAP1* mRNA prevent HeLa cells from apoptosis	Zhang C. et al., 2016
miR-92a	↑	The human tau-transgenic mice; The vGAT-ChR2 (H134R)-EYFP mice	vGATmRNA; vGAT↓(vesicular GABA transporter (vGAT).	Li et al., 2017
miR-98	↓	Hippocampal tissues of AD mice hippocampal neuronal cells	*HEY2* Notch-HEY2 signaling pathway.	Chen et al., 2019

*↑ indicates increased levels; ↓ indicates decreased levels.*

**TABLE 2:** MiRNAs in the circulation and cerebral-spinal fluid (CSF) of AD patients.

Species of miRNA	Changes	Material analyzed for miRNA	References
miR-146b-5p	↓	Peripheral blood	Wu et al., 2020
miR-15b-5p	↓	Peripheral blood	Wu et al., 2020
miR-9	↓	Peripheral blood	Souza et al., 2020
miR-1233-5p	↓	Platelet	Lee et al., 2020
miR132	↓	Neural EVs	Cha et al., 2019
miR-212	↓	Neural EVs	Cha et al., 2019
miR-339	↑	PBMC	Ren et al., 2016
miR-425	↑	PBMC	Ren et al., 2016
miR-29b	↓	PBMC	Villa et al., 2013
miR-135b	↓	Peripheral blood	Zhang et al., 2016b
miR-29c	↓	Peripheral blood	Yang et al., 2015
miR-106b	↓	Serum	Madadi et al., 2020
miR-125b	↓	Serum	Tan et al., 2014
miR-132	↑	Serum	Xie et al., 2015
miR-133b	↓	Serum	Yang et al., 2019
miR-193a-3p	↓	Serum	Cao et al., 2020
miR-19b-3p	↓	Serum	Wu et al., 2017
miR-206	↑	Serum	Xie et al., 2015
miR-222	↓	Serum	Zeng et al., 2017
miR-223	↓	Serum	Jia and Liu, 2016; Wei et al., 2018
miR-223	↓ (exosomes)	Serum	Wei et al., 2018
miR-22-3p	↑	Serum	Guo et al., 2017
miR-29c-3p	↓	Serum	Wu et al., 2017
miR-34c	↑	Serum	Shi et al., 2020
miR-384	↑ (exosomes)	Serum	Yang et al., 2018
miR-4422	↓	Serum	Hajjri et al., 2020
miR-455-3p	↑	Serum	Kumar et al., 2017; Kumar and Reddy, 2019
miR-501-3p	↓	Serum	Hara et al., 2017
miR-127-3p	↑	Plasma	Piscopo et al., 2018
miR-146a	↓	Plasma	Kiko et al., 2014
miR-181c-5p	↑	Plasma	Siedlecki-Wullich et al., 2019
miR-200a-3p	↓	Plasma	Wang et al., 2019
miR-206	↑	Plasma	Kenny et al., 2019
miR-210-3p	↑	Plasma	Siedlecki-Wullich et al., 2019
miR-342-3p	↓ (exosomes)	Plasma	Lugli et al., 2015
miR-34a	↓	Plasma	Kiko et al., 2014
miR-34a-5p	↓	Plasma	Cosin-Tomas et al., 2017
miR-34c	↑	Plasma	Bhatnagar et al., 2014
miR-545-3p	↓	Plasma	Cosin-Tomas et al., 2017
miR-92a-3p	↑	Plasma	Siedlecki-Wullich et al., 2019
miR-125b	↑	CSF	Dangla-Valls et al., 2017
miR-125b-5p	↑ (exosomes)	CSF	McKeever et al., 2018
miR-125b-5p	↓	CSF	Lusardi et al., 2017
miR-146a	↓	CSF	Kiko et al., 2014; Lusardi et al., 2017
miR-222	↑	CSF	Marchegiani et al., 2019
miR-27a-3p	↓	CSF	Sala Frigerio et al., 2013
miR-29a	↑	CSF	Muller et al., 2016
miR-34a	↓	CSF	Kiko et al., 2014
miR-451a	↓ (exosomes)	CSF	McKeever et al., 2018
miR-598	↓	CSF	Riancho et al., 2017
miR-9-5P	↓	CSF	Riancho et al., 2017
miR-let-7b	↑	CSF	Liu et al., 2018

*↑ indicates increased levels; ↓ indicates decreased levels.*

## Basic Structure and Functions of MiRNAs

MicroRNAs are small, non-coding, single-stranded RNAs approximately 22 nucleotides long. Canonical miRNA biogenesis begins with the transcription of primary miRNAs (pri-miRNAs) by RNA polymerase II. These pri-miRNAs are processed into precursor miRNAs (pre-miRNAs) by Drosha in complex with Pasha/DGCR8, and then transported from nucleus to cytoplasm. Pre-miRNAs have a hairpin loop structure recognized for cleavage by dicer, leading to the production of mature miRNAs. One strand of the mature duplex is loaded onto a member of the Argonaute (Ago) family of proteins, forming RNA-induced silencing complexes (RISCs) that mediate gene silencing by recognizing the 3′ untranslated region (3′ UTR) of target mRNAs (Schwarz and Zamore, 2002; Friedman et al., 2009; Jinek and Doudna, 2009; Ipsaro and Joshua-Tor, 2015). Of specifically note, miRNA binding with AGO can promote gene expression. For example, miR-346 recruiting AGO2 targets the 5′ UTR of amyloid precursor protein (*APP*) mRNA, which competes with the translational suppressor, the iron response protein1, thus inducing stimulative translation of *APP* mRNA (Long et al., 2019).

Besides inhibiting the expression of target genes by binding to the 3′ UTR of mRNAs, increasing evidence has indicated that miRNAs also act in a non-canonical manner by changing binding partner. MiR-181c can target mitochondrial transcription (Das et al., 2012). MiRNAs can also interact with non-Ago proteins. Research reports that miR-let-7b activates Toll-like receptors (TLRs) as a signaling molecule (Lehmann et al., 2012). Apart from binding with protein, miRNAs can synergistically interact with non-coding RNAs, including long non-coding RNAs (lncRNAs) and circular RNAs (cirRNAs). Intriguingly, miRNAs can promote gene expression, such as miR-589, binding to the promoter RNA of *COX-2* transcript, thus inducing transcriptional upregulation (Matsui et al., 2013). Another example is that miR-369 activates TNFα translation by recruiting AGO- FXR1 complex to the AU-rich elements of mRNA during G1/G0 phase. Finally, some pri-miRNAs are found to act as peptide encoding RNAs [miRNA-encoded peptides (miPEPs)], such as pri-miR-165a and pri-miR-171b (Lauressergues et al., 2015; Dragomir et al., 2018).

## MiRNAs Are Crucial for the Onset and Pathological Progression of AD

### miRNAs Are Involved in Aβ Metabolism

In 1984, George Glenner and Caine Wong found that the main component of senile plaques was a peptide of 39–43 amino acid residues, known as Aβ, thereby laying the foundation for the study of AD (Glenner and Wong, 1984). The Aβ hypothesis holds that Aβ aggregation is the causative factor in AD, leading to synaptic damage, tau protein phosphorylation, inflammation, oxidative stress, apoptosis, and eventually nerve cell damage and death (Sanabria-Castro et al., 2017). Mutations in the *APP*, presenilin 1 (*PS1*), *PS2*, and apolipoprotein E (*APOE*) genes all lead to abnormal APP processing and Aβ metabolism, resulting in Aβ deposition and subsequent neurocytotoxicity (Hardy and Higgins, 1992; Hardy and Selkoe, 2002). Other susceptibility genes for AD, such as *CLU*, *CD2AP*, *PICALM*, and *ABCA7*, affect Aβ generation and elimination (Gibson, 2010).

The *APP* gene is located in the middle segment of the long arm of chromosome 21 and contains 18 exons. APP is widely present in tissue cells throughout the body, but most abundantly expressing in neurons. APP is a type I integral inner membrane protein, comprising an intracellular domain (AICD) and an extracellular domain, and the aberrant function of APP can lead to an increase in Aβ production in AD patients (Goate et al., 1991; Rovelet-Lecrux et al., 2006). The *PS1* and *PS2* genes are located on chromosome 14 and chromosome 1, respectively, and their protein products have similar structure and function. They both contain 13 exons, and form PS proteins after transcription. PS1 possesses γ-secretase activity and participates in APP proteolysis. Mutations in *PS1* or *PS2* can affect the degradation and transport of APP, increase Aβ42 production and the Aβ42/Aβ40 ratio, and affect the interaction between tau protein and other cytoskeletal proteins, thereby attributing to the pathogenesis of AD (Kumar-Singh et al., 2006; Campion et al., 2016; Eggert et al., 2018).

*APOE* has the greatest correlation with late-onset AD (LOAD; age at onset ≥ 65 years). *APOE* has three alleles (ε*2*, ε*3*, and ε*4*) encoding APOE2, APOE3, and APOE4, respectively. APOE is a secreted glycoprotein consisting of 299 amino acids produced by astrocytes in the CNS and is associated with cholesterol transport. APOE2 and APOE3 can bind to Aβ and promote its clearance across the blood–brain barrier (BBB), while APOE4 has a relatively weak binding affinity for Aβ. The three APOE isoforms accelerate the deposition of Aβ, regulate the activity of tau-related kinases such as p35 and CDK5 through binding to receptors, and further regulate tau protein phosphorylation (Goate et al., 1991; Corder et al., 1993; Bu, 2009; Zhao et al., 2018).

MiRNAs have roles in APP degradation and Aβ metabolism through regulating the expression of related genes and associated pathways (Millan, 2017). APP degradation occurs mainly through the secretase pathway. APP is hydrolyzed by α-secretase to produce the neuroprotective soluble external functional fragments (sAPP), P3 and the AICD; in contrast, β-secretase hydrolyzes APP to produce the Aβ40 and Aβ42 forms. Aβ42 accumulates at a higher rate than Aβ40, thereby forming plaques and exerting neurotoxic effects. The γ-secretase enzyme is a key determinant of the Aβ40/Aβ42 ratio (Kaether et al., 2006; Cirrito et al., 2008; Haass et al., 2012; Roher et al., 2017).

MiRNAs can regulate the activities of key enzymes involved in APP lysis. Several miRNAs, including miR-339-5p, miR-29c, miR-15b, miR-195, and miR-124, participates in Aβ metabolism by modulating the activity of β-secretases such as BACE1 (Das et al., 2016; Selkoe and Hardy, 2016). Downregulation of miR-339-5p results in increased expression of BACE1, thus promoting Aβ deposition (Long et al., 2014). Moreover, both miR-29c and miR-135b negatively regulate BACE1 expression and show neuroprotective effects (Zhang et al., 2016b). Overexpression of hippocampal miR-188-3p reduces BACE1, Aβ, and neuroinflammation levels in APP transgenic mice (Zhang et al., 2014). AD-related ADAM metallopeptidase domain 10 (ADAM10), a member of the ADAM family of α-secretases, hydrolyzes APP to produce non-pathogenic Aβ. MiR-221 is downregulated in AD, which increases ADAM10 content (Manzine et al., 2018). MiR-140-5p is a negative regulator of ADAM10 and its transcription factor SOX2, is activated by Aβ (Akhter et al., 2018). MiRNAs have complicated interactions. MiR-107 targets BACE1 and ADAM10 also regulate APP metabolism, which suggested that single miRNA can target different genes or pathways producing additive effects (Nelson and Wang, 2010; Augustin et al., 2012; Goodall et al., 2013). BACE1 which is regulated by at least 10 more species of miRNA like miR-29c, miR-107, and miR-339-5p mentioned above. These miRNAs are both downregulated in AD showing negative correlation with BACE1. In addition, miR-221 and miR-140-5p can regulate ADAM10 negatively. However, miR-221 are downregulated in AD while miR-140-5p are upregulated, thus playing different roles. PS1 is an important component of the γ-secretory proteolytic system, and the PS1/γ-secretase system protects neurons by regulating miR-212 and PEA15 (Huang et al., 2018). Aph-1 homolog A (APH1A), a major mammalian APH1 subtype and a subunit of the γ-secretase complex. Overexpression of APH1A increases γ-secretase complex activity and consequently the levels of Aβ. MiR-151 involves in the formation of long-term episodic memory in the hippocampus by reducing the protein level of its target, APH1A (Xu et al., 2019). Increased β-secretase levels and activity elevate the levels of the AICD, which then stimulates the expression of APP and BACE1, thereby providing more substrate and enzyme for the amyloidogenic pathway. The AICD generated from the amyloidogenic pathway can translocate to the nucleus and function as a transcriptional regulator. AICD/miR-663 directly downregulates the expression of *FBXL18* and *CDK6*, which affects the growth and differentiation of neuronal cells (Konietzko, 2012; Shu et al., 2015).

MiRNAs also involves in Aβ metabolism. MiR-15/107 family members, including miR-103 and miR-107, are downregulated in AD hippocampi, and enhance the generation of Aβ and phosphorylation of APP. This increases the levels of CDK5R1/p35 and, consequently, activates cyclin-dependent kinase 5 (CDK5) and finally leads to deterioration (Jiao et al., 2016; Moncini et al., 2017). MiR-132 is significantly downregulated in the middle and late stages of AD, leading to the upregulation of inositol 1,4,5-trisphosphate 3-kinase B (ITPKB) and increased ERK1/2 and BACE1 activity in AD patients (Salta et al., 2016; Zhu Q. B. et al., 2016). MiR-330 exerts a negative regulatory effect on vav guanine nucleotide exchange factor 1 (VAV1) via the MAPK signaling pathway, which promotes Aβ generation in the AD brain (Zhou et al., 2018). Upregulation of miR-33 in AD reduces ATP-binding cassette transporter A1 (ABCA1) levels, which can regulate APOE lipidation and Aβ metabolism, thus enhancing Aβ levels (Kim et al., 2015). MiR-128 targets peroxisome proliferator-activated receptor gamma (PPARG), which promotes Aβ pathology (Muller et al., 2014; Geng et al., 2018). Some miRNAs play neuroprotective roles by reducing the secretion and toxicity of Aβ. MiR-153 reduces the expression of APP (Long et al., 2012), while miR-200b/c and miR-302 inhibit PTEN to activate Akt via the PI3K/mTOR pathway and downstream Nanog signaling, thus alleviating Aβ-induced neurotoxicity (Li et al., 2016; Wu et al., 2016; Higaki et al., 2018).

In turn, aberrantly high levels of Aβ can affect the expression of miRNAs. Overexpression of APP inhibits miR-107 (Moncini et al., 2017), and Aβ42 oligomerization can reduce the expression of miR-188-5p in hippocampal neurons (Lee et al., 2016). When cortical neurons of APP/PS1 mice are exposed to Aβ, the levels of miR-34a initially increases, and then subsequently decreases (after 48 h) (Modi et al., 2016).

### miRNAs Contribute to Abnormal Tau Protein Function

Tau is the most abundantly expressed microtubule-associated protein in neurons of the cerebral cortex, hippocampus, and axons of peripheral nerves in the human brain. There are six tau isomers in the human brain and are derived from exons 2, 3, and 10 by selective splicing (Goedert et al., 1992). Tau phosphorylation is important for its normal physiological functions, such as stabilizing the cytoskeleton, maintaining cell morphology, and ensuring intracellular transport; it also plays an important role in maintaining the protein composition of the PSD in dendritic spines in healthy neurons (Crimins et al., 2013; Iqbal et al., 2016). Aberrantly phosphorylated tau shows reduced binding affinity for microtubules, leading to tau aggregation and neurofibrillary tangle (NTF) formation, and also competitively binds to other normal microtubule-related proteins; this leads to the loss of the dynamic balance between microtubule assembly and disassembly, affects axonal transport and cell function, and results in neuronal degeneration (Sanabria-Castro et al., 2017). A variety of mechanisms, including gene mutation and an imbalance in tau protein-mediated regulation of enzyme function, lead to abnormal tau protein phosphorylation (Alonso et al., 2018; Davila-Bouziguet et al., 2019; Penke et al., 2019).

MiRNAs can not only directly affect tau protein synthesis, such as miR-219 directly targeting *MAPT* (Santa-Maria et al., 2015), but can also affect tau phosphorylation by regulating the activity of the relevant enzymes. GSK3, PKA, and CDK5 protein kinases can reveal or hide phosphorylation sites to synergistically adjust tau protein phosphorylation, primarily on serine and threonine residues, while phosphatases such as PP2A, PP2B, and PP1 dephosphorylate tau protein at multiple sites to varying degrees (Lee et al., 2011). CDK5, a proline-directed serine/threonine kinase, can regulate tau phosphorylation. P35/P25 are activators of CDK5, and calpain (CAPN)-induced cleavage of p35, which generates p25, gives rise to the aberrant activation of CDK5 and promotes tau hyperphosphorylation (Lopes and Agostinho, 2011). MiR-124-3p, which reduces in AD, inhibits the translation of *CAPN1* mRNA, prevents the conversion of p35 to p25 and the subsequent formation of the p25/CDK5 complex, and reduces abnormal tau phosphorylation (Zhou et al., 2019). Overexpression of miR-125b leads to the upregulation of the p35, CDK5, and p44/42 MAPK (Erk1/2) signaling pathways, while the phosphatases DUSP6 and PPP1CA and the antiapoptotic factor Bcl-W are downregulated as direct targets of miR-125b, which promotes tau hyperphosphorylation (Banzhaf-Strathmann et al., 2014; Ma et al., 2017). GSK3 is also an important kinase for tau protein phosphorylation, and miR-219-5p downregulates GSK3 to inhibit tau phosphorylation in AD (Li et al., 2019).

Additional mechanisms are reported via which miRNAs can affect tau phosphorylation. MiR-132/212 plays an important role in memory formation and maintenance (Hernandez-Rapp et al., 2015). Its downregulation affects the balance of S-nitrosylation and induces tau phosphorylation and aggregation in a NOS1-dependent manner *in vivo* (Pichler et al., 2017; Salta and De Strooper, 2017; Wang et al., 2017). MiR-322, a rodent homolog of human miR-424, promotes tau phosphorylation by negatively regulating brain-derived neurotrophic factor (BDNF)-TrkB receptor activation (Zhang et al., 2018). MiR-146a upregulation results in tau hyperphosphorylation in neurons through modulation of the ROCK1/PTEN signaling pathway (Wang et al., 2016). MiR-138 overexpression induces tau hyperphosphorylation by targeting the RARA/GSK3β pathway, increasing tau phosphorylation at Thr231, Ser396, and Ser404 (Wang X. et al., 2015). MiR-106b inhibits Aβ42-induced tau phosphorylation at Tyr18 by targeting Fyn (Liu et al., 2016), while miR-137 exerts inhibitory actions on tau phosphorylation by suppressing *CACNA1C* expression (Jiang et al., 2018). Changes in tau protein levels also affect miRNA expression. Tau accumulation increases miR-92a levels in AD, thereby inducing anxiety through the miR-92a/vGAT/GABA signal in the mouse (Li et al., 2017).

### miRNAs Regulate Synaptic Plasticity

Synapse formation is the basis of neural signal transduction, while synaptic plasticity is the basis of learning and memory. Memory impairment in AD patients is recognized to be a result of abnormal synaptic plasticity. Overexpression of miR-34c in hippocampal neurons influences AD pathogenesis by negatively regulating dendritic length and spine density (Kao et al., 2018). An increase in the level of soluble Aβ enhances glutamate release and excitatory toxicity (Edwards, 2019). Synapses are vulnerable to Aβ-induced neurotoxicity, and miRNAs regulates Aβ-mediated synaptic toxicity and plasticity. The CAMKK2/AMPK/Tau pathway is a key mediator of Aβ42 oligomer-mediated synaptic toxicity (Mairet-Coello et al., 2013). MiR-431 protects synapses and neurites from Aβ-induced toxic effects via the Wnt/β-catenin signaling pathway (Ross et al., 2018). MiR-188-5p can also alleviate Aβ42-mediated synaptic damage and dysfunction (Lee et al., 2016). The N-methyl-D-aspartate receptor (NMDAR) is an ion channel protein localized in the postsynaptic membrane and an important “molecular switch” for learning and memory. The excitation of NMDAR leads to a continuous increase in Ca^2+^ concentration, impairment of long-term potentiation (LTP), toxic damage, and loss of synapses. NMDA receptors drive glutamate-induced neuroexcitotoxicity, and a variety of factors, including p38 kinase, contribute to Aβ-mediated neurotoxicity (Hardingham and Bading, 2010). BDNF also contributes to the regulation of synaptic function. Inhibiting miR-132 reduces the increase in BDNF-dependent postsynaptic protein expression. MiR-132/212 family members play important roles in neural function and synaptic plasticity, and are continuously downregulated in early AD (Kawashima et al., 2010; Pichler et al., 2017). A potential target of miR-132 in cholinergic neurons may be a regulator of cholinergic transmission and synaptic plasticity, which may indirectly promote Aβ42 production and lead to cholinergic neurodegeneration (Zhu L. et al., 2016). MiR-200c is also downregulated in the frontal and temporal lobes of AD brains, exerting a protective effect against endoplasmic reticulum stress (ERS)-induced loss of cholinergic neurons (Wu et al., 2016).

### miRNAs Participate in Neuronal Growth, Differentiation, and Apoptosis

Neural stem cells (NSCs) are characterized by their capacity to proliferate and differentiate into multiple neuronal cell types, including neurons, astrocytes, and oligodendrocytes. How to promote the differentiation of NSCs into neurons as a means of replacement therapy in AD is currently the subject of intensive research efforts. MiRNAs modulate the growth, development, maturation, and differentiation of neurons to varying degrees. MiR-142a-5p, miR-146a-5p, miR-155-5p, and miR-455-5p are upregulated in the AD brain and regulate neuronal function, and may also have a role in brain development and neurodegeneration (Arena et al., 2017; Sierksma et al., 2018). MiR-135a targets the thrombospondin 1 (*THBS1*) 3′ UTR, thereby promoting angiogenesis (Ko et al., 2015). MiR-511 increases neuronal differentiation and development as a functional regulator of *FKBP5* in primary neurons (Zheng et al., 2016). MiR-302/367 induces the reprogramming of reactive astrocytes to neurons, contributing to neural repair, and may represent a potential therapeutic strategy to restore learning and memory (Ghasemi-Kasman et al., 2018).

In the AD brain, miRNAs play a two-way regulatory role in apoptosis, one of the main causes of neuronal loss in AD patients. On the one hand, miRNAs can promote apoptosis. For example, the downregulation of miR-512 in AD leads to an imbalance in proapoptotic/antiapoptotic factors, thereby promoting apoptosis and further deterioration (Mezache et al., 2015). Aβ also induces the downregulation of miR-34a, enhances the expression of cyclin-D1, and promotes the neuronal cell cycle through the MEK/ERK signaling pathway, which leads to apoptosis (Modi et al., 2016). MiR-146a inhibits *LRP2* translation, which also leads to cell apoptosis (Zhang B. et al., 2016). On the other hand, miRNAs can also inhibit apoptosis. MiR-19 is a key component of the miR-17-92 cluster and inhibits aluminum-induced neuronal apoptosis (Zhu M. et al., 2016). MiR-214-3p negatively regulates the expression of ATG12 by targeting its 3′ UTR, inhibits autophagy, and reduces the levels of apoptosis in hippocampal neurons (Zhang et al., 2016a). Increased expression of miR-4487 decreases Aβ-induced apoptosis in neurons (Hu et al., 2018). Furthermore, miR-98 reduces Aβ production, inhibits the Notch signaling pathway, and suppresses the apoptosis of hippocampal neurons, thereby promoting their survival (Chen et al., 2019). MiR-124-3p attenuates tau phosphorylation-induced neuronal apoptosis by targeting the caveolin-1/PI3K/Akt/Gsk3β pathway (Kang et al., 2017). MiR-125b regulates inflammatory factors and oxidative stress through SphK1, thereby mediating the growth and apoptosis of neuronal cells (Jin et al., 2018). MiR-603 is an intronic miRNA of *KIAA1217*, a gene that is highly expressed in the human brain and elicits protective effects on neuroanl cells (Zhang C. et al., 2016).

### miRNAs Mediate Immuno-Inflammatory Responses in AD

Immuno-inflammation is one of the pathological hallmarks of AD. Microglia and astrocytes both participate in the physiological function of central neuritis. Microglia, an immune effector cell in the brain, protects neurons from neuronal loss by eliminating harmful substances, but also exerts toxic effects on neurons through the activity of proinflammatory factors. Microglia neuroinflammation may act as an early trigger or as a sustained vulnerability factor that aggravates pathophysiological processes driving AD, leading to neurol loss. Aβ and oxidative stress can activate microglia and astrocytes, leading to Ca^2+^ influx and mitochondrial damage in synapses, followed by neurodegeneration. However, microglia effectively clears damaged synapses to prevent further extensive axonal damage (Kuchibhotla et al., 2008), and also releases cytokines and chemokines through a process known as “synaptic pruning,” assists and guides the process of neuronal differentiation, and mitigates Aβ-mediated toxic damage (Nayak et al., 2014; Salter and Beggs, 2014).

Numerous SNPs and rare coding variants in immune-related genes thought to be involved in microglial function have been identified as risk factors for AD in whole-genome sequencing and GWAS analyses, including *TREM2, CR1, SHIP1, BIN1, CD33, PICALM, CLU*, and the *MS4A* gene cluster (Gibson, 2010; Rosenberg et al., 2016; Bis et al., 2018). *TLR*-associated gene polymorphisms have been linked with susceptibility to LOAD (Sohrabifar et al., 2015). MiRNAs may activate TLRs and play a role in neuroinflammation under certain conditions (Bryniarski et al., 2015). TREM2 is an immunoglobulin superfamily receptor found in microglia, and mutations in the *TREM2* gene increase the risk of LOAD (Cheng-Hathaway et al., 2018; Parhizkar et al., 2019). MiR-34a targets 299 nucleotides of the 3′ UTR of the *TREM2* mRNA, resulting in the downregulation of *TREM2* and microglia phagocytosis (Bhattacharjee et al., 2016). Knocking out *TREM2* reduces neuroinflammation in AD mice (Leyns et al., 2017).

MiRNAs can play both protective and pathogenic roles by influencing neuroinflammatory responses through inflammation-associated cytokines. MiR-9, miR-34a, and miR-155 exhibit an anti-inflammatory effect through the modulation of downstream targets of proinflammatory mediators in the brain, including TNF receptor-associated factor 6 (TRAF6) and interleukin 1 receptor-associated kinase 1 (IL1R-AK1). Complement factor H (CFH) is a suppressor of the inflammatory response, and miR-125, miR-146a, and miR-155 enhance harmful CFH-induced proinflammatory events in AD, which may be associated with oligomeric Aβ induced inflammatory responses (Millan, 2017). MiR-139 has a negative regulatory effect on responses to proinflammatory stimuli, and prevent AD progression through the regulation of cannabinoid receptor type 2 (CB2)-mediated neuroinflammation (Tang et al., 2017). Prostaglandin E2 (PGE2) is a key mediator of the inflammatory response. PGE2 regulates CCAAT/enhancer-binding protein delta (CEBPD) in astrocytes through the EP4 receptor and protein kinase A, and CEBPD activation is associated with AD. Following PGE2 treatment, CEBPD induces miR-135a activation in astrocytes to inhibit THBS, suggesting that the CEBPD/miR135a/THBS1 axis may be a therapeutic target for the treatment of AD (Ko et al., 2015).

## Spatial and Temporal Expression of MiRNAs in AD

As outlined above, miRNAs participate in the onset and pathological progression of AD. The expression levels of miRNAs show spatial and temporal differences in AD patients. MiRNAs like miR-9, miR-124, miR-125b, and miR-132, are expressed specifically in the CNS (Millan, 2017), and their dysregulation is associated with neurodegenerative diseases, such as AD.

MiRNA expression differs between gray matter and white matter in AD, although relatively few miRNAs are specifically altered in the white matter of AD brains. For example, the levels of miR-132 and miR-212 in AD brain reduce to different degrees in gray matter and white matter and more prominent in gray matter compared with healthy subjects (Pichler et al., 2017). Different miRNA species have different physiological functions, and their expression and distribution in AD also differ. The level of miR-107 in the hippocampus and temporal lobe decreases, while that of miR-146a increases during AD (Millan, 2017). Moreover, even the same miRNA shows different expression patterns between cerebral regions. MiR-29c is upregulated in the hippocampus of mice in the early stages of AD, but is significantly downregulated in the cortex (Zong et al., 2015). The difference can be partly explained by different cell composition and functions, as well as the properties of the different types of miRNA and differences in research methods (Smith et al., 2015; Salta et al., 2016). Gray matter and white matter are important components of the CNS. Gray matter comprises mostly neurons, astrocytes, endothelial cells, microglia, and relatively few oligodendrocytes, while white matter functions mainly in conduction (Wang et al., 2010). The cerebral cortex, closely related to learning and memory, is composed of gray matter. Moreover, the neural-specific pathological changes in AD, including amyloid plaques and NFTs, are primarily found in gray matter (Cech and Steitz, 2014). The pathological changes occurring in AD show temporal continuity, beginning in the entorhinal cortex at the base of hippocampus, and subsequently spreading to frontal lobe, temporal lobe, and occipital cortex with continued disease development, leading to impaired learning and memory function, as well as personality changes. This may also underlie the spatial and temporal changes of miRNA profiles. One example is that hippocampal MiR-128 elevates in the middle stage of AD, whereas decreases in the late stage (Muller et al., 2014; Geng et al., 2018). The expression of miR-132 in the nucleus basalis of Meynert is fairly stable in the early stage of AD, but is significantly downregulated in late stage (Zhu Q. B. et al., 2016). MiR-212 expression is similar to that of miR-132 (Pichler et al., 2017).

## miRNAs Help With the Diagnosis of AD

AD is a pathophysiological continuum and can be divided into three stages according to clinical and pathological changes: the early preclinical stage, mild cognitive impairment (MCI), and subsequent dementia. Two commonly applied diagnostic criteria, NIA-AA and IWG-2, both recommend the application of a variety of biomarkers and methods for the stratification, classification, and differential diagnosis of AD. Most biomarkers and methods focus on the late stage of the disease, and can be summarized as follows: (1) Neuropsychological tests: Cognitive assessments such as the Mini-Mental State Examination (MMSE) can be used for early diagnosis to quantitatively assess the severity of cognitive impairment and record cognitive changes over time; however, this method depends on factors such as the patient’s education level and familiarity with the test, which limits its specificity and sensitivity. (2) Neuroimaging examination: Magnetic resonance imaging (MRI) and fluorodeoxyglucose (FDG)–positron emission tomography (PET) can be used to observe the pathological changes and functional abnormalities that can occur without obvious cognitive impairment, including medial temporal lobe atrophy and metabolic abnormalities. Although this method can be practical, it has important limitations in terms of time and cost. (3) Other biomarkers: Aβ_1__–__40_, Aβ_1__–__42_, total tau (t-tau), and phosphorylated tau (p-tau) proteins in the cerebrospinal fluid (CSF) are currently the best biomarkers for clinical research and the monitoring of AD. However, CSF acquisition requires a lumbar puncture, which is invasive and not easily accepted by the patients. Additionally, the detection of disease-causing genes also can be used. AD diagnosis still lacks efficient, simple, and inexpensive biomarkers, especially for the early stages of the disease.

In this respect, miRNAs have several advantages over classical biomarkers. Several studies have shown that specific species of miRNAs detected in the biofluid of AD patients are consistent with the observed pathological changes (Keller et al., 2016; Swarbrick et al., 2019; Takousis et al., 2019; Wiedrick et al., 2019). MiRNAs in serum, plasma, or CSF show great stability when they are enwrapped in liposomes or bound to lipoproteins, which prevents their degradation and allows them to withstand severe environmental conditions (van den Berg et al., 2020). Moreover, miRNAs can be easily obtained and quantified using real-time PCR, next-generation sequencing (NGS), or microarray. Some of the findings are summarized in Table 2.

The main potential applications are as follows: (1) A biomarker for the diagnosis of AD. A systematic review and meta-analysis of 10 studies comprising 770 AD patients and 664 normal controls indicated that miRNAs display excellent diagnostic performance, showing an overall sensitivity of 0.80 (95% CI: 0.75–0.83), a specificity of 0.83 (95% CI: 0.78–0.87), and a diagnostic odds ratio of 14 (95% CI: 11–19) (Zhang et al., 2019). Serum miRNA biomarkers related to AD prognosis show consistency with neuropsychological and neuroimaging assessments, and plasma levels of miR-34a-5p and miR-545-3p have potential as biomarkers in early AD; however, further large-scale research is still needed to confirm this (Cheng et al., 2015; Cosin-Tomas et al., 2017). Serum miR-133b levels in AD patients are positively correlated with the simple intelligence status test score. The area under the ROC curve of miR-133b in the diagnosis of AD was 0.907, with 1.7 as the critical value, with a sensitivity of 90.8% and a specificity of 74.3% (Yang et al., 2019). There was a significant positive correlation between the serum level of miR-193a-3p and the MMSE score in AD patients (Cao et al., 2020). Although some studies showed connection between miRNAs and cognition tests, there are inadequate studies directly focusing the combination of miRNAs and MMSE, as well as other clinical diagnosis including CSF Aβ and tau, PET imaging. MiR-193a-3p has potential for use as a new biomarker to distinguish AD patients from healthy people. The serum concentrations of miR-222, miR-29c-3p, and miR-19b-3p also have potential as biomarkers for AD (Wu et al., 2017; Zeng et al., 2017), as do miR-455-3p, miR-29a, miR-107, miR-106a-5p, and miR-324-3p (Wang T. et al., 2015; Muller et al., 2016; Yilmaz et al., 2016; Cai et al., 2018; Kumar and Reddy, 2018). Combining between two and four miRNAs can distinguish AD from controls with an accuracy of 75–82% (Lusardi et al., 2017). Discrimination analysis using a combination of miR-100, miR-103, and miR-375 could detect AD in CSF by positively classifying controls and AD cases with 96.4 and 95.5% accuracy, respectively (Denk et al., 2015). The combination of serum miR-223 and miR-125b levels provided improved sensitivity/specificity for AD prediction than either miRNA alone (Jia and Liu, 2016). A 54 months study found that an AD-specific 16-miRNA signature can predict AD with a sensitivity and specificity of 87 and 77%, respectively. Each participant were assessed by cognitive assessments and Aβ neuroimaging during this study, and those AD participants with normal clinical manifestations diagnosed by Aβ neuroimaging suggested a higher risk of progression toward AD (Cheng et al., 2015). Changes in plasma APOE, miR-107, and miR-650 levels may be a marker of neurodegeneration during AD associated with amyloid metabolism and cell cycle disorders (Prendecki et al., 2019). (2) A predictor of the conversion from mild cognitive impairment (MCI) to AD. Approximately 10–15% of MCI patients enter the dementia stage each year, and amnestic MCI (aMCI) patients may have a higher risk of developing AD (Giau et al., 2019). A 5-year follow-up study showed that an increased serum level of miR-206 may be a potential predictor of aMCI-to-AD conversion. There was a positive correlation between serum miR-206 levels and the rate of progression from aMCI to AD (Kenny et al., 2019). However, these results need to be confirmed in more studies. Bioinformatic analysis indicated that the serum levels of miR-519d-3p could be the bridge regulator between MCI and AD; however, this requires further verification (Tao et al., 2020). Plasma miR-92a-3P, miR-181c-5p, and miR-210-3p levels are significantly upregulated in MCI and AD patients. Patients with MCI progressing to AD had higher plasma levels of these miRNAs (Siedlecki-Wullich et al., 2019). MiR-135a and miR-384 levels were increased and miR-193b levels were decreased in patients with AD and MCI, while the combination of these three miRNAs could predict the risk of MCI onset and conversion to AD (Giau et al., 2019). However, no study has evaluated the diagnostic performance of differentially expressed miRNAs between MCI/AD patients and healthy controls (Pena-Bautista et al., 2019). Indeed, the lack of recognized and reliable reference genes in the analysis of miRNAs in patients with MCI seriously hinders the analysis and limits research on circulating miRNAs (Piscopo et al., 2019). (3) A tool to differentiate AD from other neurodegenerative diseases. The expression levels of exosomal miRNA-384 in the serum of AD and non-AD patients differ significantly. In addition, the serum level of exosomal miR-384 has potent differential diagnostic ability for AD and Parkinson’s disease dementia (PDD), as well as for AD and vascular dementia (VaD), with sensitivity/specificity indices of 97.2%/100% and 99.1%/100%, respectively (Yang et al., 2018). Using this 12-miRNA signature, the differentiation of AD from other neurological diseases is possible with accuracies of between 74 and 78%. The differentiation of the other CNS disorders from controls yields even higher accuracies (Leidinger et al., 2013). Gender differences have been found in the analysis of plasma miRNA in patients with frontotemporal dementia (FTD) (Grasso et al., 2019), but not in those with AD. Indeed, miRNA biomarkers show considerable inconsistencies between studies, and results are hard to reproduce. Studies with the same sample source and subjects of similar demographic background can generate different or even contradictory results (Jain et al., 2019; Pena-Bautista et al., 2019). This can be explained by the different procedures and methods of sample collection, miRNA detection, and data analyses. Differences in miRNA isolation procedures, cell contamination and hemolysis, quantitative methods, reference genes, and sample quality control can all affect the final results (Piscopo et al., 2019). For example, platelets are rich in miRNAs and release large amounts of miRNAs into the circulation during the coagulation process, leading to differences between serum and plasma miRNA concentrations (Wang et al., 2012). Additionally, miRNAs are more stable in platelets than in the corresponding plasma and serum samples, as indicated by the higher miRNA concentration in platelets (Lee et al., 2020). Similarly, CSF contamination with blood cells is a major confounding factor when analyzing CSF-derived miRNAs. In such a scenario, the analysis of cell-free CSF-derived exosomes could be superior to total CSF analysis and may also explain some of the discrepancies among the results (Müller et al., 2016). In addition, miRNA profiles vary with the biofluid or the exosome. A major reason for this variation is the selective transportation of miRNAs. Specifically, mature miRNAs can be actively sorted into microvesicles (MVs) by specific proteins (membrane-localized proteins, RNA-binding proteins) and then released into the biofluid (Li et al., 2018; Groot and Lee, 2020). Neuron-derived exosomes can cross the blood–brain barrier (BBB) and transfer their cargo to the CSF (Barbagallo et al., 2020). MiRNAs are also passively released from apoptotic bodies or platelets during coagulation. Biomarkers in exosomes are suggested to have higher diagnostic efficiency and be of better quality than those in biofluid (Nie et al., 2020). Notably, different approaches used for exosome purification may also lead to differences in results (Lee et al., 2019).

## Summary and Outlook

MiRNAs display wide distribution patterns throughout the CNS. They mainly interact with non-coding sequences of target messenger RNAs, and play important regulatory roles in the development, maturation, differentiation, and gene expression of neuronal cells. MiRNAs with different physiological functions are differentially expressed between brain regions, and influence various aspects of AD pathogenesis through different pathways. MiRNAs can collectively exert more pronounced effects. Several studies have noted that one miRNA acts on hundreds of targets, while multiple miRNAs also coordinate to act on the same mRNA sequence, resulting in an intricate network (Ameres and Zamore, 2013; Barry, 2014). These data suggest that, although the expression of individual miRNAs may have specific effects, the overall effect of miRNAs will not be fully understood until the global miRNA expression patterns in the brain have been elucidated. How to identify simple and effective pathways in this complex network, and then guide the theory and practice, is a future subject requiring intensive investigation. Although the mechanisms underlying the effects of miRNA dysregulation in AD are increasingly identified, research is still in the early stages. Most studies are relatively scattered. The breadth and depth of related studies need to be expanded to further screen the key mechanisms involving in the interactions between miRNAs and AD to provide new insights for the study of pathogenesis, to identify effective indicators and targets for diagnosis, and to administrator as a cognition-improving treatment.

In this review, we summarized existing evidence about miRNAs serving as diagnostic biomarkers in AD. The use of miRNAs as AD biomarkers still faces many challenges, even though a substantial number of miRNAs have been identified that have relatively high efficiency, specificity, and sensitivity for diagnosing AD. The following points need to be further clarified: (1) whether the changes in miRNA content in different brain regions, cerebrospinal fluid, and serum are related and whether the changes are AD-specific; (2) some miRNAs may show opposing trends during different stages of AD, and how to effectively divide the boundaries remains a major challenge; (3) because miRNAs exert a multitarget effect, and many key factors of AD are influenced by more than one species of miRNA: this is exemplified by BACE1 which is regulated by at least 10 more species of miRNA. Hence, it is important to effectively identify the individual roles of specific miRNAs, as well as the collective role of multiple miRNAs in AD. In addition, procedures for sample collection, miRNA detection, and data handling need to be standardized to increase the repeatability of results. Finally, the use of a combination of multiple miRNAs as markers, or combining miRNAs with other biofluid biomarkers, may perform better in the diagnosis, differentiation, and prediction of AD. Large sample trials are required to reach a robust conclusion (Figure 2).

## Author Contributions

WW wrote the manuscript. Z-YW, L-NM, and T-TZ assisted in the manuscript writing. YC and HL assisted in ideas and modification of the manuscript. All authors contributed to the article and approved the submitted version.

## Conflict of Interest

The authors declare that the research was conducted in the absence of any commercial or financial relationships that could be construed as a potential conflict of interest.
